# A Comparative Study of Nozzle/Diffuser Micropumps with Novel Valves

**DOI:** 10.3390/molecules17022178

**Published:** 2012-02-22

**Authors:** Kai-Shing Yang, Tzu-Feng Chao, Ing Youn Chen, Chi-Chuan Wang, Jin-Cherng Shyu

**Affiliations:** 1 Green Energy and Environment Research Laboratories, Industrial Technology Research Institute, Hsinchu 31040, Taiwan; Email: ksyang@itri.org.tw; 2 Department of Mechanical Engineering, National Yunlin University of Science and Technology, Yunlin 64002, Taiwan; Email: ycshsummer@yahoo.com.tw (T.-F.C.); cheniy@yuntech.edu.tw (I.Y.C.); 3 Department of Mechanical Engineering, National Chiao Tung University, Hsinchu 30010, Taiwan; 4 Department of Mechanical Engineering, National Kaohsiung University of Applied Sciences, Kaohsiung 80778, Taiwan; Email: jcshyu1207@hotmail.com

**Keywords:** micro pump, diffusers, nozzles, enhancement, flow rate

## Abstract

This study conducts an experimental study concerning the improvement of nozzle/diffuser micropump design using some novel no-moving-part valves. A total of three micropumps, including two enhancement structures having two-fin or obstacle structure and one conventional micro nozzle/diffuser design, are made and tested in this study. It is found that dramatic increase of the pressure drops across the designed micro nozzles/diffusers are seen when the obstacle or fin structure is added. The resultant maximum flow rates are 47.07 mm^3^/s and 53.39 mm^3^/s, respectively, for the conventional micro nozzle/diffuser and the added two-fin structure in micro nozzle/diffuser operated at a frequency of 400 Hz. Yet the mass flow rate for two-fin design surpasses that of conventional one when the frequency is below 425 Hz but the trend is reversed with a further increase of frequency. This is because the maximum efficiency ratio improvement for added two-fin is appreciably higher than the other design at a lower operating frequency. In the meantime, despite the efficiency ratio of the obstacle structure also reveals a similar trend as that of two-fin design, its significant pressure drop (flow resistance) had offset its superiority at low operating frequency, thereby leading to a lesser flow rate throughout the test range.

**Table:** 

List of symbols			
*A*	Amplitude (m)	*θ*	Opening angle (deg)
*f* **	Frequency (Hz)		
*Q*	Flow rate (m^3^/s)	*η*	ratio of the loss coefficient of nozzle and diffuser
*ū*	Mean velocity (m/s)	*ξ*	Total pressure loss coefficient
*V*	Voltage (V)	*ρ*	Density (kg/m^3^)
*W*	Throat width (m)	*ΔP*	Pressure drop (Pa)
Subscripts and Superscripts			
*diff*	Diffuser	*nozzle*	Nozzle

## 1. Introduction

Recently, microscale pumping had received significant attention in industrial, medical, and biological applications such as lab-on-a-chip, fuel cells, high flux electronic cooling and biochemistry [1,2]. Normally microvalves are employed in the micropumps to achieve a higher efficiency. In practice, three different microvalve structures have been widely used in the design of micropumps, including active valve [3], passive check valves [4], and valve-less structure [5]. Other considerations for micropumps, depending of specific applications, are drug compatibility, small size, power consumption, and flow rate controllability over a wide range of external conditions [6]. 

Among the types of microvalve structures, active valves with actuators are often adopted in micro-pump design. The design features easier flow controllability over a wide range of operating conditions at the expense of larger size and additional power consumption. There is no additional power consumption for the passive valve design, but it offers lower controllability. In the meantime, either active or passive valves are prone to clogging, wear, and fatigue which are always major issues for micro-pump applications. To tackle this problem, the novel concept of a valve-less diffuser pump was first proposed by Van De Pol [7]. Stemme and Stemme [5] later took the concept a step forward with a workable and practical micropump. 

The valve-less diffuser pump consists of two diffuser elements connected to a pump chamber with an oscillating diaphragm. Unlike those using passive check valves or active check valves, the design uses nozzles/diffusers as flow directing elements. Hence wear and fatigue in the valves are eliminated since no moving parts are encountered with the diffuser elements, thereby the risk of clogging is drastically reduced. The key components of the nozzle/diffuser micropump are the flow directing diffuser elements. Hence, many subsequent investigations have been made to understand the flow characteristics inside the nozzle/diffuser elements and to seek an optimal design for such micropump applications. Studies have involved detailed investigations either via experiments [8], numerical simulations [9,10], flow visualization [11], or combined efforts simultaneously using numerical simulations and experimental data [12,13] were also performed. Most of the relevant studies concerning nozzle-diffuser micropumps are based on steady state operation, although the pumping action relies on dynamic actuation. To clarify the influence of the dynamic effects of the nozzle-diffuser micropump, some studies focus on the transient behaviors of nozzle/diffuser [14,15], actuator, and coupling [16,17].

Despite its simple and robust nature, the nozzle/diffuser micropump suffers from low efficiency. The concept of using a double chamber to improve the performance of valveless micropumps was proposed [18,19]. However, this type of micropump normally consists of complex structures having more actuators, thereby leading to higher cost and stability problems. In this regard, it is of crucial importance to seek some form of augmentation to improve the efficiency for this kind micropump. However, the published literature about micro nozzles/diffusers is mainly focused on the manufacturing technology as well as its performance [8,20,21] or on simulating the performance of micropumps with micro nozzle/diffuser valves [19,22], yet there is very little attention paid towards performance improvements using enhanced structures. For the performance of mircovalves alone, Yang *et al*. [23] characterized and analyzed the performances of four micro nozzles/diffusers having enhancement structures with a conventional micro nozzle/diffuser valve. They reported a maximum improvement of the loss coefficient ratio of about 16%. The objective of the present study was to incorporate the associated concepts into micropumps, examining the performance of the micropumps subject to these enhancement structures.

## 2. Results and Discussion

**Figure 1:**
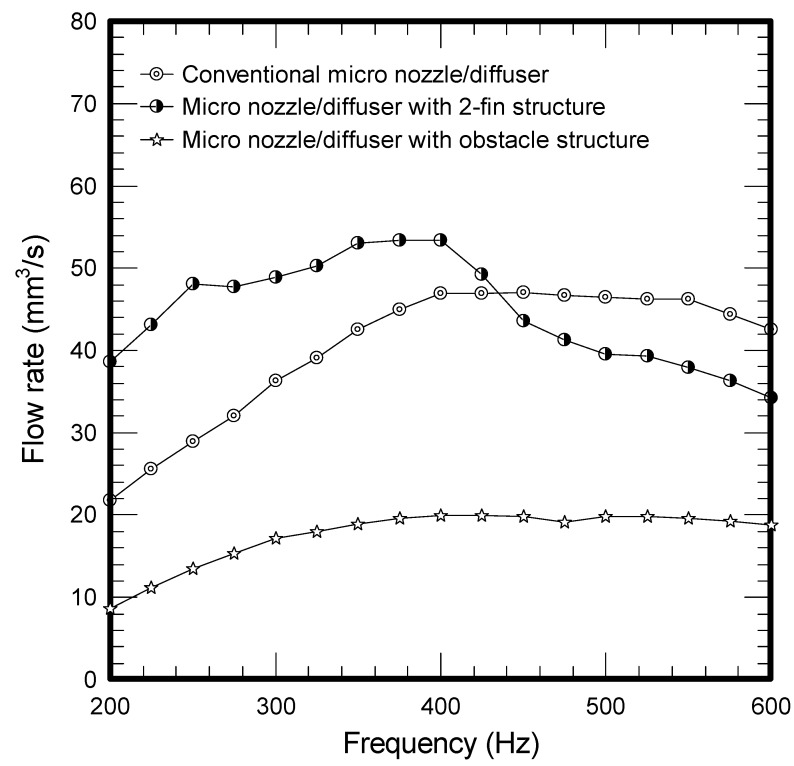
Frequency *vs*. flow rate for micropumps.

**Figure 2:**
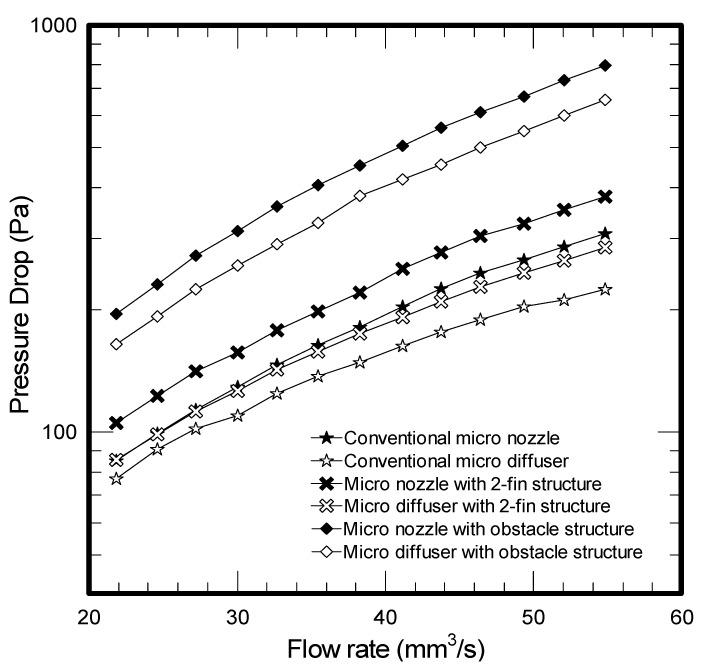
Flow rate *vs*. pressure drop for micro nozzle/diffuser.

**Figure 3:**
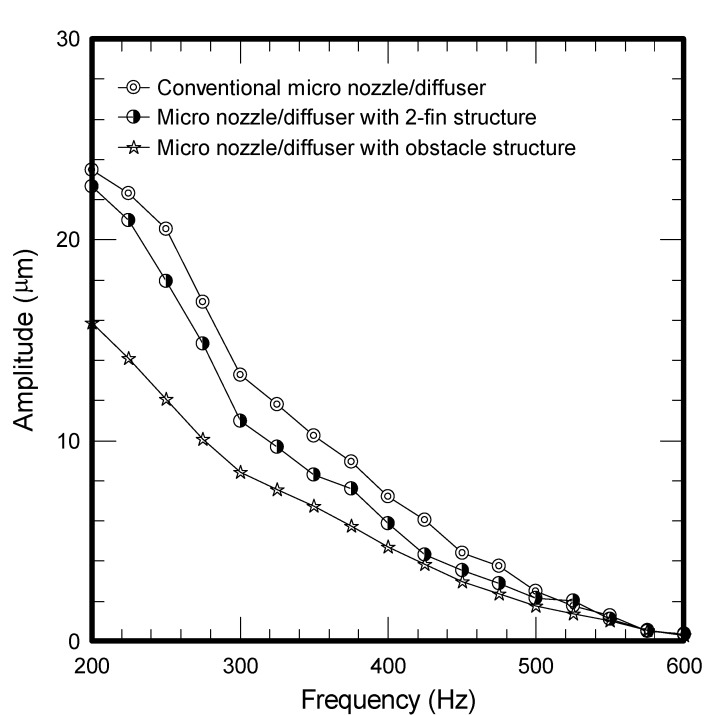
Frequency *vs*. amplitude for tested micropumps.

The results of mass flow rate *vs*. frequency for all the tested micropumps at a fixed voltage of 50 V are plotted in Figure 1. As depicted in the figure, the flow rate for all three micropumps is firstly increased with the rise of frequency and peaks at a frequency around 375 ~ 425 Hz. The corresponding maxima for the conventional, two-fin, and obstacle structure are 47.07 mm^3^/s, 53.39 mm^3^/s and 19.79 mm^3^/s, respectively. However, the obstacle structure gives the least flow rate among the tested samples. In the meantime, the flow rate for the two-fin structure exceeds that of conventional design when the frequency is below 425 Hz, yet the trend is reversed when the frequency surpasses 425 Hz. Apparently, the performance of micropumps is related to the micro structures of the diffuser element. Firstly, the presence of enhanced structure like the present two-fin structure or obstacles will give rise to more pressure drops. This can be made clear from Figure 2 where the obstacle causes a significant pressure drop, followed by the 2-fin structure and conventional design. The much higher pressure drop leads to a sharp rise of flow resistance, thereby reducing the vibrating amplitudes as shown in Figure 3. Accordingly, the smallest flow rate is encountered for the obstacle structure. In this situation, the maximum amplitude of the conventional micro nozzle/diffuser and added obstacle structure are 23.49 μm and 15.85 μm.

However, as opposed to the added obstacle structure in micro nozzle/diffuser, despite the pressure drop for the 2-fin structure still exceeds that of conventional one, the flow rate for the two-fin design is superior to the conventional one when the operating frequency is below 425 Hz. For a further comparison of the performance for the test samples, the pressure drops are then in terms of dimensionless efficiency ratio *vs*. the flow rate. 

**Figure 4:**
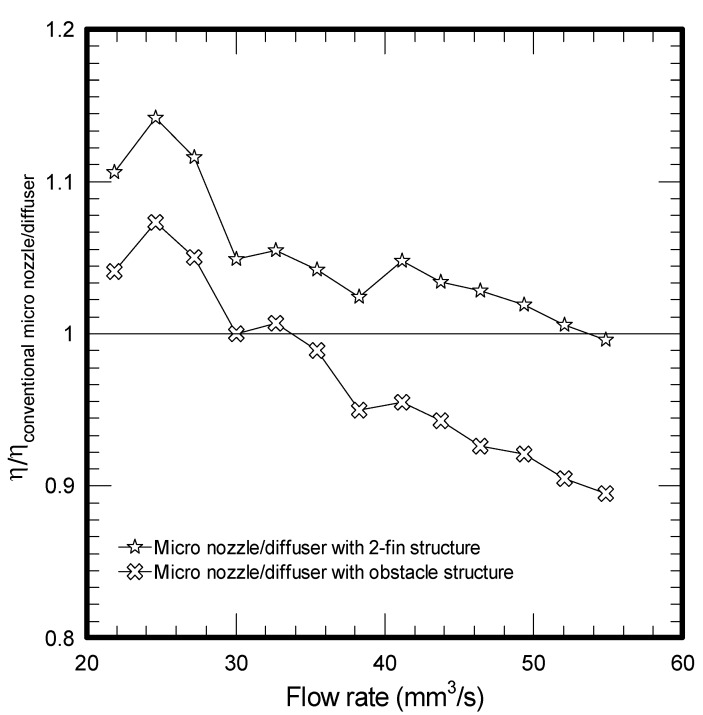
Flow rate *vs*. the efficiency ratio between conventional micro nozzle/diffuser.

Test results are shown in Figure 4, where the ordinate of the figure is η/η_conventional micro nozzle/diffuser_. A value above unity indicates that the efficiency ratio for the enhanced design exceeds that of conventional nozzle/diffuser at the same flow rate. 

The results shown in this figure denote that the micro nozzle/diffuser with added fins substantially outperforms the conventional design in the low flow rate region. The maximum efficiency ratio improvement is about 15%. As a consequence, the added two-fins structure in micro nozzle/diffuser shows a higher mass flow rate when the frequency is below 400 Hz. However, in a conventional micro nozzle/diffuser, the loss coefficient for the nozzle at the exit is higher. This is because the free jet flow is accompanied by some additional pressure recovery for the diffuser, leading to a higher efficiency in the higher flow rate region 11. In the meantime, as shown in Figure 2, the added fin offers an additional pressure drop as compared to the conventional design. This becomes more pronounced when the flow rate is increased. In summary, these effects result in a higher mass flow rate for the conventional design at the higher operating frequency.

## 3. Experimental Setup

**Figure 5:**
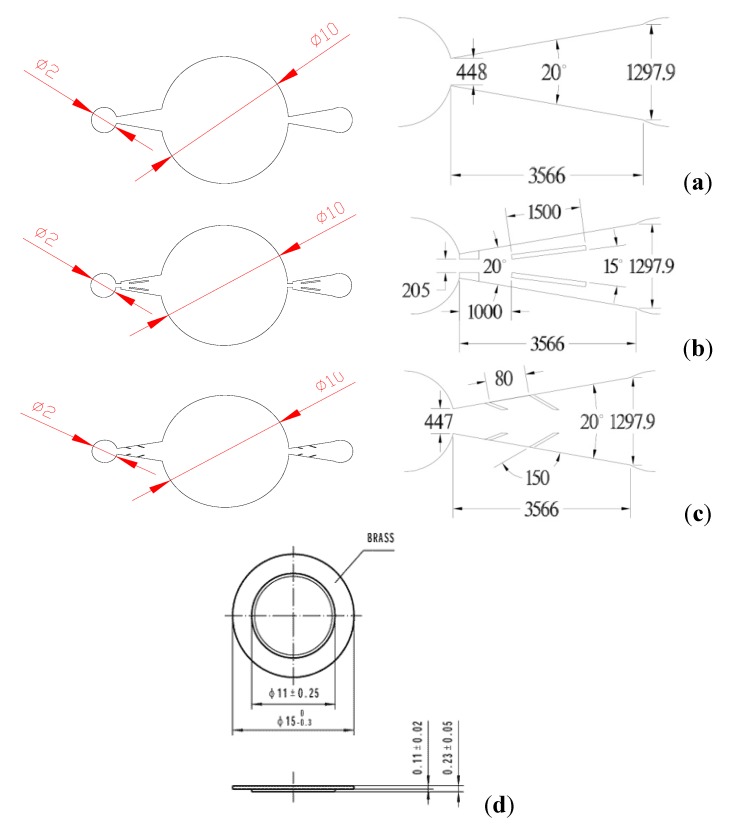
Detailed geometry of the test micro nozzle/diffuser and piezoelectric disks.

Before finalizing the manufacturing of a real micropump, we had conducted prior designs of the micro nozzles/diffusers using Computational Fluid Dynamics (CFD) to obtain a better design. A total of three types of micro nozzle/diffuser are implemented in the micropumps. The geometries of the test micro-nozzle/diffuser structure and their detailed dimensions are shown in Figure 5. 

**Figure 6:**
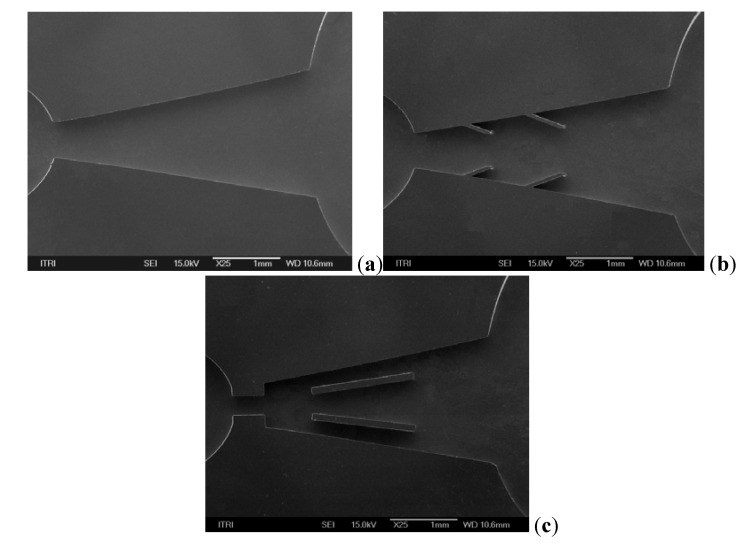
The SEM photo of obstacle tested sample.

Figure 5a denotes the conventional design with an opening angle of 20°, Figure 5b is the two-fin structure with the same opening angle, and Figure 5c is the obstacle structure. Detailed geometry of the piezoelectric disk (Kepo Electronic, KPTG-15T-6.0A1) is shown in Figure 5d. The test samples were fabricated using the deep reactive ion etching (DRIE) technique. SEM photos showing the fabricated samples are given in Figure 6a–c. The inlet and outlet hole are drilled using a laser machining on the glass wafer. Finally the silicon wafer is anodically bonded to the glass wafer. 

The test samples of micro nozzle/diffuser were then placed in a test rig to examine their performance. The present experimental setup to measure the total pressure drop across the nozzle/diffuser is the same as the one used in the study by Yang *et al*. [23]. The total pressure drop Δ*P* of micro nozzle/diffuser is often expressed in terms of the pressure loss coefficient ξ, *i.e*.:


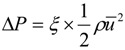


The most common way to evaluate the micropump performance is via the efficiency ratio of the nozzle/diffuser element as follows: 


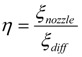


**Figure 7:**
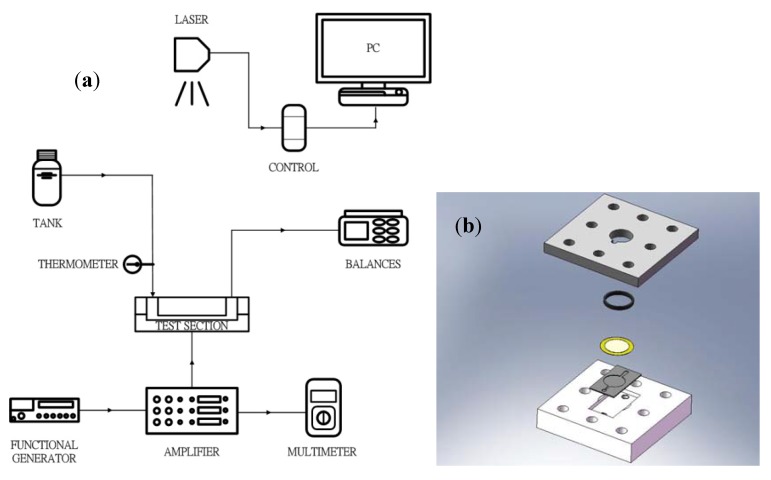
Schematic diagram of the experimental setup of (**a**) micropump (**b**) test section.

The schematic of the mircopump testing facility is shown in Figure 7a, and the test section is shown in Figure 7b. A function generator (GW Instek, GFG-8216A with controlled frequencies ranging from 0.3 Hz to 3 MHz; Taipei, Taiwan) is used to generate control signal for the piezo element. The generated signal is further amplified by a SVR500-3 amplifier (Piezomechanik Gmbh; Munich, Germany). The corresponding voltage is from −100 to 500 V. The amplitude of the piezo membrane can be measured by a laser displacement sensor (Keyence LK-G30; Osaka, Japan) with a measurement range of ±5 mm and an accuracy of ±0.01 μm). The mass delivered by the micropump is measured by a precision electronic balance (A&D GF-2000, with minimum detectable mass weight of 0.01 g; Tokyo, Japan). The measured mass weight is then divided by the collected time to give the mass flow rate. 

## 4. Conclusions

This study characterizes and analyzes the performance of micro pumps using two types of enhancement structures, including two-fin and obstacle structures in comparison with the conventional micro nozzle/diffuser design with fixed geometric dimensions such as length, widths, depth, and angles. The pressure drops across the designed micro nozzles/diffusers are found to be considerably increased when the obstacle or added fin structure are employed. The resultant maximum flow rates are 47.07 mm^3^/s and 53.39 mm^3^/s for a conventional micro nozzle/diffuser and added two-fin structure, respectively, at a frequency of 400 Hz. It is found that the flow rate for the two-fin design is higher than the conventional one when the frequency is below 425 Hz whereas the trend is reversed when the frequency is above 425 Hz. This is because the maximum efficiency ratio improvement for the added two-fin structure is appreciably higher than the conventional one at a lower operating frequency but the trend is gradually reduced when the operating frequency is further increased. In the meantime, despite the fact the efficiency ratio of the obstacle structure also reveals a similar trend as that of the two-fin design, its significant pressure drop (flow resistance) offsets its superiority at low operating frequency, thereby leading to a smallest flow rate throughout the test range. In this situation, the maximum amplitude of the conventional micro nozzle/diffuser and added obstacle structure are 23.49 μm and 15.85 μm, respectively. This study examined two specific augmented structures applicable to the nozzle/diffuser micropump, and the obtained results are quite promising. For optimizing the proposed enhanced structure, it is recommended that further investigations on two aspects are performed. Firstly, the associated optimal designs characterizing dimensions such as length, widths, depth, and angles are important due to their influence on the rectification properties and are recommended for future study. Secondly, future research to clarify the dynamic effects of nozzle/diffuser micropump with enhancement are also essential.
